# Immunity of human epithelial ovarian carcinoma: the paradigm of immune suppression in cancer

**DOI:** 10.1186/1479-5876-11-147

**Published:** 2013-06-13

**Authors:** Vincent Lavoué, Aurélie Thédrez, Jean Levêque, Fabrice Foucher, Sébastien Henno, Vincent Jauffret, Marc-Antoine Belaud-Rotureau, Veronique Catros, Florian Cabillic

**Affiliations:** 1 Lady Davis Institut, Jewish General Hospital, McGill University, Montreal, QC H3T 1E2, Canada; 2 Faculty of Medicine, University of Rennes 1, Rennes, France; 3 Liver Metabolisms and Cancer, Inserm, UMR991, Rennes, France; 4 Department of gynecologic surgery, Teaching Hospital of Rennes, Rennes, France; 5 Department of pathology, Teaching Hospital of Rennes, Rennes, France; 6 Department of cell biology, Teaching Hospital of Rennes, Rennes, France; 7 Service de chirurgie gynécologique, CHU Hôpital Sud, 16 Bd de Bulgarie, Rennes, 35 000, France

**Keywords:** Ovarian carcinoma, Immunity, Immune suppression

## Abstract

Epithelial ovarian cancer (EOC) is a significant cause of cancer-related mortality in women, and there has been no substantial decrease in the death rates due to EOC in the last three decades. Thus, basic knowledge regarding ovarian tumor cell biology is urgently needed to allow the development of innovative treatments for EOC. Traditionally, EOC has not been considered an immunogenic tumor, but there is evidence of an immune response to EOC in patients. Clinical data demonstrate that an antitumor immune response and immune evasion mechanisms are correlated with a better and lower survival, respectively, providing evidence for the immunoediting hypothesis in EOC. This review focuses on the immune response and immune suppression in EOC. The immunological roles of chemotherapy and surgery in EOC are also described. Finally, we detail pilot data supporting the efficiency of immunotherapy in the treatment of EOC and the emerging concept that immunomodulation aimed at counteracting the immunosuppressive microenvironment must be associated with immunotherapy strategies.

## Introduction

Epithelial ovarian cancer (EOC) is the fifth most common cancer among women and the fourth most common cause of cancer-related death among women in developing countries [1]. The prognosis is poor, with a 5-year survival rate of 30%. The majority of patients relapse within 16–18 months following the end of treatment and die from the disease despite response to first-line therapy consisting of debulking surgery and chemotherapy [2,3]. 15% of patients die within the first year. No substantial decrease in the death rate occurred in the last three decades. Thus, there is an urgent need for basic knowledge of ovarian tumor biology for the development of innovative EOC treatments.

Unlike melanoma or renal and hematologic tumor diseases, EOC is not considered to be immunogenic. However, there is evidence of an immune response against EOC in patients [4]. Experimental data show that the inflammatory microenvironment of EOC prevents the maturation of myeloid cells, favors regulatory cell development and restrains the cytotoxic activity of effector lymphocytes, leading the tumor to escape from the immune system and triggering cancer progression [5]. Treatments such as chemotherapy with paclitaxel/carboplatin and debulking surgery are traditionally considered to negatively impact the immune system during EOC [6]. However, recent data challenge this concept and highlight the major role of immune response in EOC. Indeed, aforementioned treatments were shown to modulate the host response and to decrease the immunosuppression [7,8]. Thus, immunotherapies aimed at increasing the host immune response or decreasing immunosuppression were tested in preclinical and clinical studies and are emerging as potential strategies to enhance classical EOC treatments.

In this article, we present an overview of the current understanding of the immune response and immune suppression in EOC. The immunological role of chemotherapy and surgery is highlighted, and pilot data supporting the efficiency of immunotherapy in EOC treatment are reviewed.

### Evidence of an immune response in EOC

EOC expresses or overexpresses tumor-associated antigens (TAA), i.e. antigens (Ag) acquired by tumor cells in the process of neoplastic transformation that can elicit a specific T-cell immune response by the host. In 1993, EOC ascites were found to contain CD8^+^ T-cells capable of recognizing HER2/neu-positive tumor cells [9]. 5 to 66% of EOC exhibit this EGFR-related glycoprotein that activates signaling pathways involved in cellular proliferation [10,11]. Many other TAA were described in EOC, such as folate receptor(FR)-α [12], epithelial cell adhesion molecule (EpCAM) [13], human epididymis protein 4 [14], p53 [15], mucin-like MUC16 (CA125) and MUC1 (CA15.3) [16] and TAA of the cancer-testis group [17,18]. Tumor-reactive T-cells and antibodies (Ab) directed against TAA were detected in the peripheral blood of patients with advanced-stage disease at the time of diagnosis [15,19], and tumor-reactive T-cells were isolated from tumors or ascites [20].

**Figure 1:**
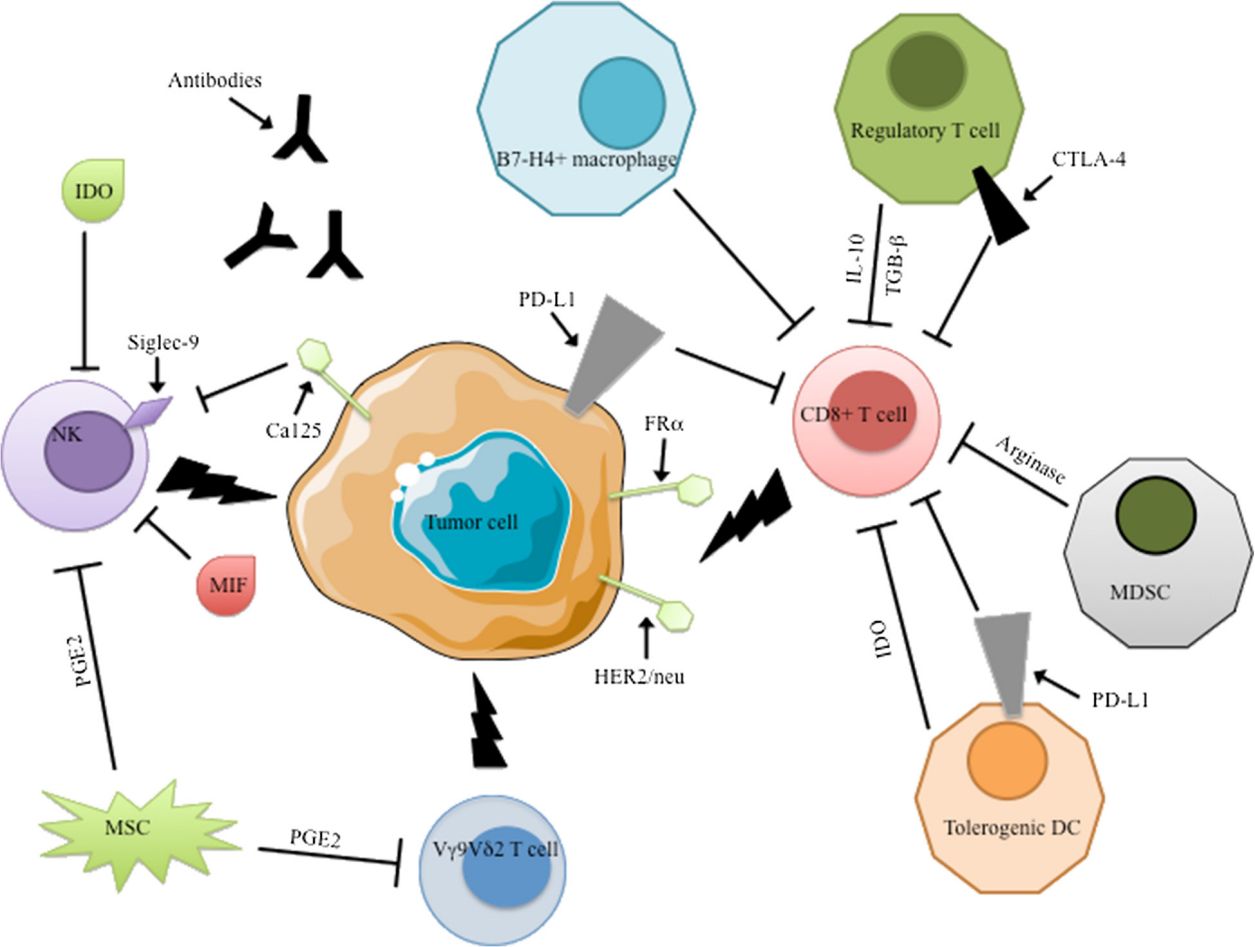
**Immune network in EOC. **EOC is immunogenic and expresses tumor-associated antigens such as HER2/neu, CA125 and Folate Receptor α. Various immune effectors such as CD8^+ ^T-cells, NK-cells and Vγ9Vδ2T-cells can attack tumor cells, but immunosuppressive crosstalk counteracts the functions of these effector cells. Treg, tolerogenic DC, MDSC, B7-H4^+ ^TAM and non-immune cells such as mesenchymal stem cells and tumor cells themselves halt antitumor activities through cell-cell contacts (CA125/siglec pathway, PD-1 and CTLA-4 immunosuppressive checkpoints) or the production of soluble factors (IL-10, TGF-β, PGE2, MIF, arginase-1, and IDO).

Furthermore, there is clinical evidence for the role of immunosurveillance against EOC. The detection of intraepithelial tumor-infiltrating lymphocytes (TIL) correlates with clinical outcome. Zhang et al. detected CD3^+^ TIL in 102/186 frozen specimens from patients with stage III/IV EOC [21]. The five-year progression-free survival rates were 31.0% and 8.7% for patients with and without TIL, respectively. The presence of TIL correlated with progression-free survival in multivariate analysis (p < 0.001) [21]. Recently, other studies confirmed that the CD3^+^ TIL count is a significant prognosis factor in EOC (Table 1) [22-32]. High frequencies and activity levels of immune effector cells such as CD8^+^ T-cells, Natural Killer(NK)-cells and Vγ9Vδ2T-cells are correlated with positive clinical outcomes for EOC patients [33,34]. Thelper(Th)-17 cells, a recently discovered T-lymphocyte subset, were found in proportionally higher number in EOC microenvironment in comparison with other immune cells [35,36]. In EOC patients, Th17 levels in the tumor correlated positively with Th1-cells, cytotoxic CD8^+^ T-cells and NK-cells and Th17 levels in ascites correlated positively with patient survival [35]. Intriguingly, Th17 were reported to promote either tumor cell growth or antitumor response and their role in cancer development is currently under debate [37]. Finally, in addition to TIL, the number of peripheral blood immune cells, e.g. NK-cells, is also correlated with survival in EOC [33]. All these results support the existence of immunosurveillance in EOC (Figure 1).

**Table 1:** Clinical arguments for the immunoediting hypothesis in epithelial ovarian carcinoma

**Authors**	**Year**	**Findings**
**Spontaneous anti-tumor response**		
Zhang L et al. [21]	2003	Association between intraepithelial T-cell infiltration (TIL CD3^+^) and patient survival
Raspollini NR et al. [22]	2005	Association between intraepithelial T-cell infiltration (TIL CD3^+^) and patient survival (plus chemotherapeutic response)
Sato E et al. [23]	2005	Association between intraepithelial T-cell infiltration (TIL CD8^+^) and patient survival
Hamanishi J et al. [24]	2007	Association between intraepithelial T-cell infiltration (TIL CD3^+^) and patient survival
Clarke B et al. [25]	2008	Association between intraepithelial T-cell infiltration (TIL CD8^+^) and patient survival (only for high grade serous EOC, but not for endometrioïd or mucinous EOC)
Shah CA et al. [26]	2008	Association between intraepithelial T-cell infiltration (TIL CD8^+^) and optimal debulking surgery
Tomsova M et al. [27]	2008	Association between intraepithelial T-cell infiltration (TIL CD3^+^) and patient survival
Callahan MJ et al. [38]	2008	Association between intraepithelial T-cell infiltration (TIL CD8^+^) and patient survival
Han LY et al. [28]	2008	Association between intraepithelial T-cell infiltration (TIL CD3^+^ and CD8^+^) and patient survival
Stumpf M et al. [29]	2009	Association between intraepithelial T-cell infiltration (TIL CD3^+^ and CD8^+^) and patient survival
Leffers N et al. [30,39]	2009	Association between intraepithelial T-cell infiltration (TIL CD8^+^) and patient survival
Milne K et al. [31]	2009	Association between intraepithelial T-cell infiltration (TIL CD3^+^ and CD8^+^) and patient survival
Adams SF et al. [32]	2009	Association between intraepithelial T-cell infiltration (TIL CD3^+^ and CD8^+^) and patient survival
Kryczek I et al. [35]	2009	Association between intraepithelial T-cell infiltration (TIL CD4^+^ with IL-17 secretion) and patient survival
**Tumor immune evasion**		
Curiel TJ et al. [36]	2004	Inverse association between survival and intratumoral regulatory T cells (CD4^+^CD25^+^FoxP3^+^)
Wolf D et al. [40]	2005	Inverse association between survival and intratumoral regulatory T cells (FoxP3^+^)
Dong HP et al. [41]	2006	Inverse association between survival and intratumoral NK (CD3^-^ CD16^+^) or B cells (CD19^+^)
Kryczek I et al. [42]	2007	Inverse association between survival and intratumoral B7-H4^+^ macrophage or regulatory T cells (FoxP3^+^)
Hamanishi J et al. [24]	2007	PD-L1 expression by tumor predicts low T-cell infiltration
Buckanovitch RJ et al. [43]	2008	Endothelin B receptor (ET_B_R) expression restricts T-cell infiltration and predicts poor survival
Labidi-Galy SI [44,45]	2011	Inverse association between survival and intratumoral pDC (CD4^+^, CD123^+^, BDCA2^+^)

PD-1: programmed cell death 1; PD-L1: PD-1 ligand 1; ET_B_R: endothelin B receptor; pDC: plasmacytoïd dendritic cells.

### Immune escape in EOC

The tumor immunosurveillance concept was postulated in the 1960s by Burnet and Thomas, who proposed that the immune system patrols the body to recognize and destroy host cells that become cancerous and that the immune system is responsible for preventing cancer development [46]. This concept was then replaced by the cancer-immunoediting hypothesis, in which the immune system shapes tumor immunogenicity with three successive phases: elimination, equilibrium and escape [47].

Immune escape in EOC involves several mechanisms that implicate tumor, immune and stromal cells. Ovarian tumor cells escape immune recognition by downregulating surface molecules involved in Ag presentation, such as β2-microglobulin and Major Histocompatibility Complex (MHC) [28]. Similarly, the downregulation of MHC class I-related chain A (MICA) expression impedes the detection of tumor cells by innate cytotoxic effector cells through the engagement of the NKG2D-activating receptor [48,49]. Additionally, ovarian tumor cells overexpress molecules that counteract the cytotoxic activities of immune cells: CA125 binds the NK-cell inhibitory receptor (KIR) siglec-9, thereby protecting themselves from NK-mediated lysis [50,51]; the macrophage migration inhibitory factor (MIF) downregulates NKG2D-activating receptor expression on NK-cells [52]. Furthermore, engagement of programmed death-1 (PD-1) on CD8^+^ T-cells by programmed death-1 ligand-1 (PD-L1) expressed by ovarian tumor cells impairs the effector functions of these lymphocytes [24,53]. Wide panel of cancers, including EOC, were also shown to express indolamine-2,3-dioxygenase (IDO), an intracellular enzyme that catalyzes the rate-limiting step in the metabolism of the essential amino acid tryptophan [54,55]. IDO is a beneficial host mechanism regulating immune responses in various contexts such as pregnancy, transplantation or infection. It was proposed to elicit feedback process, therefore preventing deleterious consequences of excessive immune responses. However, this endogenous mechanism is hijacked by tumors to establish immunotolerance to tumor antigens [56,57].

Immune cells also play a major role in the immune escape in EOC [58]. The EOC-specific recruitment of CD4^+^CD25^+^FoxP3^+^ regulatory T-cells (Treg), tolerogenic dendritic cells (DC), B7-H4^+^ tumor-associated macrophages (TAM) and myeloid-derived suppressor cells (MDSC) fosters immune privilege and predicts reduced survival in EOC (Table 1) [23,36,44,59-62]. Accumulation of Treg is now well documented in various tumors including EOC [23,36,38-41]. CCR4 chemokine receptor expression confers to Treg higher capacity than effector T-cells to infiltrate the tumor in response to CCL22 chemokine produced by either tumor cells or TAM [36]. In addition, Treg could originate from *in situ* expansion. In that setting, ICOS-ligand costimulation provided by plasmacytoid DC (pDC) was recently highlighted as a prominent signal triggering *in situ* Treg expansion in some tumors, including EOC [63,64]. At last, *de novo* conversion of FoxP3^-^ cells into Treg was shown to occur in the tumor as a consequence of TGF-β stimulation or IDO induction [65,66]. Treg mainly mediate immunosuppression through cell-cell contacts with DC or effector cells or by the secretion of immunosuppressive cytokines, including IL-10, IL-35 and TGF-β [67]. Treg notably contribute to DC tolerization, thereby further reducing the effector T-cell activation and proliferation. Interestingly, association of tumor regulatory T-cells with high hazard ratio for death and decreased survival times is currently well documented in EOC [23,36,42]. Besides Treg, DC are instrumental in establishing immunosupression in cancer. While DC were initially recognized as the primary orchestrators of the immune response, their role in the immunotolerance is now well established [68]. Importantly, both conventional myeloid DC (cDC) and pDC are characterized by high plasticity [69]. Consequently, their immune properties could be modulated by environmental stimuli and tumors may benefit from this Achille’s heel to induce DC tolerization and to reduce the adaptive immunity to tumor antigens. Accordingly, studies showed that the EOC microenvironment converts DC toward an immunosuppressive phenotype [70]. In a mouse model of EOC, Scarlett et al. showed that the DC phenotype controls EOC progression. Indeed, the switch of infiltrating-DC from activating to regulatory phenotype coincides with rapid tumor progression to terminal disease [62]. The role of pDC in EOC immunity was proposed by Zou et al. that evidenced the recruitment of pDC in response to stromal-derived factor-1 (SDF-1/CXCL-12) secretion by EOC [71]. The accumulation of pDC within the EOC was shown to be associated with shorter progression-free survival [44]. Tolerogenic DC may exert profound immunosuppressive effects on effector lymphocytes. Alteration of the IFN-α production by pDC was recently documented in EOC [44]. Moreover, through PD-L1/PD-L2 expression, DC can engage the PD-1 inhibitory pathway, thus inhibiting lymphocyte proliferation and effector functions [72,73], inducing tumor-specific T-cell apoptosis [74] and promoting the differentiation of CD4^+^ T-cells into Treg [75]. Tolerogenic DC can also turn-down the immune response through the induction of IDO activity that inhibits CD8^+^ T-cell proliferation [76] and decreases NKG2D expression on NK-cells [77]. As aforementioned for DC, the tumor microenvironment also strongly polarizes the macrophage differentiation and gives rise to TAM [37]. B7-H4^+^ macrophages, a subset of TAM, was shown to suppress TAA-specific T-cell immunity [60]. An inverse correlation was evidenced between the intensity of B7-H4 expression on macrophages in EOC and patient survival [42]. Moreover, average 5-year survival rate was found significantly higher in EOC patients with low densities of TAM than in patients with increased TAM populations [78]. At last, MDSC are immature myeloid cells with immunosuppressive properties that were evidenced in both mouse model of EOC and EOC patients [61,79,80]. MDSC exhibit increased level of arginase-1 (ARG-1) and inductible Nitric Oxide Synthase (iNOS) activities. Deprivation of L-Arginine in the tumor microenvironment is emerging as a key immunosuppressive mechanism. It leads to CD3-zeta chain downregulation, thereby inhibiting effector T-cell activation [81]. Increased levels of NO, along with reactive oxygen and nitrogen species, disrupt signaling through the IL-2 receptor [82] and alter Ag recognition by nitrating the TCR [83]. Moreover, MDSC were shown to facilitate effector T-cell conversion into Treg [84] and to inhibit intratumoral migration of CD8^+^ effectors because of the nitration of CCL2 chemoattractant [85].

Third player in tumor escape is the stromal cell population. Overexpression of the endothelin-B receptor by tumor endothelial cells inhibits concurrent ICAM-1 expression, thereby impairing the ICAM-1/LFA-1-mediated transmigration of leukocytes [86]. Overexpression of the endothelin-B receptor is associated with the absence of TIL and short survival time in EOC patients [43]. Furthermore, stromal cells may provide chemoattractants for the immune cells e.g. SDF-1/CXCL12 that recruits pDC [71]. They are also able to secrete soluble immunosuppressive factors e.g. prostaglandin-E2 (PGE2) which is produced by mesenchymal stem cells (MSC).

Finally, the EOC microenvironment is characterized by the presence of numerous immunosuppressive soluble or cellular factors (IL-10, TGF-β, PGE2, MIF, HLA-G, IDO, arginase-1, PD-L1, B7-H4 and Fas-ligand), which can originate from various sources, including tumor, immune and stromal cells [87-91]. PGE2 can be secreted by both MSC and EOC tumor cells. Of note, overexpression of COX-2, an inducible enzyme that triggers PGE2 synthesis, by ovarian tumor cells correlates with resistance to chemotherapy and poor prognosis [92]. PGE2 inhibits NK and γδ T-cell cytotoxicity [45,93,94] and induces the differentiation of CD4^+^ T-cells into Treg [95]. Similarly, IDO is expressed in ovarian tumor cells and tumor-infiltrating DC [54,55,96]. IDO expression was reported in 43% of analyzed EOC tissues (83/192) [97]. Moreover, its expression was correlated with worse patient survival [54,55] and with enhanced peritoneal tumor dissemination [55,98]. IDO is currently thought as one of the main factors that contribute to tumor-induced immunosuppression by depleting tryptophan from the microenvironment and producing tryptophan metabolite kynurenine. Depletion of tryptophan is sensed by GCN2 kinase pathway driving effector T-cell anergy and apoptosis [99]. Effects of kynurenine are mediated by the aryl hydrocarbon receptor transcription factor that induces increased survival and motility in cancer cells while favoring Treg expansion and suppressive effects in effector T-cells [100,101].

Thus, regulatory cells, along with soluble and cellular immunosuppressive factors, create a tolerogenic microenvironment in EOC that compromises the antitumoral immune response [89]. These EOC immunosuppressive networks characterize the “cancer immunoediting” concept, which emphasizes a dynamic process of interaction between cancer and the host immune system [47] (Figure 1).

### Modulation of the immune response against EOC with debulking surgery or chemotherapy

Conventional EOC treatment uses debulking surgery and systemic chemotherapy. Surgery decreases the tumor burden and removes poorly vascularized tissues while cytotoxic drugs eradicate residual tumor cells [7,102,103]. Little information is available regarding the impact of surgery on the immunological status in EOC patients. Major surgery would induce immunosuppression because of the downregulation of T helper(Th)-1 response [6,104]. However, there is some evidence that tumor debulking reduces tumor-induced immunosuppression in EOC [7,105]. Napoletano et al. demonstrated that surgery significantly decreases the proportions of Treg and naive CD4^+^ T-cells while significantly increasing the ratio of CD8^+^ T-cells/Treg and the proportions of effector T-cells among the peripheral blood mononuclear cells [7]. Moreover, surgery significantly increases IFN-γ secretion by peripheral CD8^+^ T-cells and reduces the IL-10 immunosuppressive factor concentration in the serum [7]. Thus, cancer immunosuppression is partially reversible, and acquired immunity is enhanced by tumor debulking surgery in EOC [7].

Regarding chemotherapy, the frequent induction of lymphopenia suggests that this treatment may be immunosuppressive. However, recent data indicate that immunity plays a major role in the therapeutic mechanisms associated with chemotherapy [106,107]. Accordingly, in advanced-EOC patients treated with platinum-based chemotherapy, an optimal tumor debulking outcome was more frequent when CD3^+^ TIL are present [21]. In addition, paclitaxel or cisplatin used in EOC cause the upregulation of mannose-6-phosphate receptor expression on murine tumor cells. This upregulation sensitizes tumor cells to granzyme-B protease released by cytotoxic T-lymphocytes [108]. Paclitaxel can also stimulate the proliferation of T-cells and enhance the cytolytic activity of NK-cells in models of breast cancer [106,109]. Moreover, in advanced EOC, successful chemotherapy was shown to be associated with improved functions and increased proportions of CD8^+^ effector T-cells [7,8]. Furthermore, chemotherapy decreases immunosuppression by reducing the number of circulating Treg observed after neoadjuvant chemotherapy in EOC [7]. Some antitumor agents can also trigger immunogenic tumor cell death, causing the cancer cells to expose or secrete immunogenic signals that trigger an anticancer immune response. Of note, not all types of chemotherapy, but oxaliplatin and 3/25 tested anthracyclines, elicit immunogenic cell death [110-112]. Altogether, these data provide evidence that debulking surgery and chemotherapy may restore, by direct and indirect effects, the equilibrium phase or the elimination phase in tumors that escaped initial immunosurveillance [106].

### Immunotherapy in EOC: how to counteract immunosuppression?

Preclinical and preliminary clinical studies aimed at proving the immunotherapy concept in EOC were initiated by using monoclonal Ab (mAb), vaccinations or adoptive T-cell transferts [113-115]. The majority of these studies were uncontrolled phase I/II studies, with small sample sizes and heterogeneous inclusion criteria (recurrent or chemotherapy-refractory diseases) disrupting the comparisons and the identification of the best strategy.

Several mAb targeting TAA were tested in EOC [116]: anti-CA125 oregovomab and abagovomab [117-119]; anti-HER-2/neu trastuzumab and pertuzumab [10,120]; anti-FR-α farletuzumab α [121], anti-EpCAM catumaxomab [122] and anti-Tag72 B72.3 [123]. All these mAb demonstrated adequate safety and tolerability but failed to demonstrate clear clinical benefits, even when an immunological response was evidenced [114,115]. Active immunotherapy by vaccination based on peptides or cellular approaches were also evaluated. Clinically tested peptides include NY-ESO-1 [124,125], p53 [126], HER2-neu [127] and multiple constructed-peptides (HER2-neu/MAGE-A1/FRα [128] and MUC-1/carcinoembryonic antigen [129]). In addition, cell vaccines include DC pulsed with ARNm (FRα [130]), peptides (HER2-neu, MUC1 [131]), autologous tumor Ag [132] or whole tumor cell lysate [133]. Vaccine therapies were well tolerated and demonstrated immunological responses, but provided only minor clinical benefits. Of note, these studies enrolled low numbers of patients and have generally not yet evolved past phase I/II studies. A third immunotherapy strategy is adoptive T-cell therapy, which uses cytotoxic lymphocytes with natural or engineered reactivity against cancer cells. Cytotoxic lymphocytes are generated *in vitro* and then transferred back into the patient to elicit cytotoxic responses against the patient’s own tumor cells. Only five phase I/II EOC studies, which enrolled few patients (<20), are available [134-138]. They used either TIL or peripheral autologous T-cells and were well tolerated; unfortunately, only modest clinical benefits were demonstrated. Thus, to date, results of these trials are disappointing, regardless of the strategy [115]. However, these trials may suffer from some pitfalls. First, they often enrolled patients with recurrent or refractory-chemotherapy diseases, i.e. patients at terminal stages of the disease, with strong immunodepression. It is likely that enrollment of patients at earlier stages of disease could be more successful. Secondly, all these trials focused on the recognition and killing of tumor cells and neglected to consider the immunosuppressive impact of the tumor microenvironment. Thus, improvement of theses immunotherapies is needed. For example, chimeric antigen receptor(CAR)-modified T cell therapy is highly promising [139,140]. CAR T-cells could be engineered to only express the downstream pathway of activating receptors. This refined adoptive therapy skips inhibitory signals expressed by the tumor environment. In addition, use of adjuvant drugs targeting immunosuppressive cells or soluble/cellular immunomodulatory factors could be the key to fully unleash the potential of immunotherapy by breaking peripheral tolerance.

Below, we review some immunomodulatory tools already in clinical use or likely to be assessed in the near future, that interact with the immunosuppressive factors found in the EOC microenvironment.

First approach may consist in depleting the host of the regulatory cells or in limiting their recruitment within the tumor. Treg depletion may be achieved using low-dose cyclophosphamide which prevents, under incompletely understood mechanism, Treg development and functionality [141,142]. An alternative strategy uses the expression by Treg of the IL-2 receptor alpha (CD25). Recombinant fusion protein of IL-2 and diphtheria toxin (Ontak^®^, Eisai) was tested in EOC patients and showed effective depletion of circulating Treg [143]. Moreover, in patients with metastatic breast cancer, the anti-CD25 mAb daclizumab (Zenapax^®^, Roche) demonstrated selective T-lymphocyte killing properties, allowing Treg depletion for several weeks [144]. However, it is unclear if Treg depletion occurs at EOC locations (solid tumor, malignant ascites) and results in tumor regression [143-145]. Moreover, as effector cells also express CD25, anti-CD25 mAb may also induce unwanted depletion of effector cells [146]. In addition, blocking the ICOS-pathway could inhibit the pDC-triggered proliferation of Treg within the tumor [64]. However, as ICOS pathway also favors the differentiation of T helper(Th)-17 cells which might either promote tumor growth or antitumor response [35,37,147-150] careful preclinical investigations of ICOS inhibitors (314.8 mAb) is needed [63].

The role of chemoattractants in the recruitment of immune cells also gives a great opportunity to reduce the infiltration of regulatory cells within the tumor [151]. First candidates are under investigation. CCR4 antagonists were shown to block the recruitment of Treg instructed by CCL22 and CCL17 and to favor the induction of antigen-specific CD8^+^ T cell response after vaccination [152]. Similarly, Bindarit^®^ that inhibits CCL2 synthesis and therefore restricts the recruitment in the tumor of immature myeloid cells, was shown to induce tumor regression in prostate and breast cancer animal models [153]. Regulatory cell depletion could also be achieved by improving the maturation of immature myeloid cells [154] using all trans retinoic acid [155] or ultra-low non-cytotoxic doses of paclitaxel (chemo-immunomodulation) [156].

Another attractive approach is the use of either antagonists of immune-repressor molecules or agonists of immune-activating receptors [157]. Checkpoint blockade receptors comprise CTLA-4, PD-1 and NK inhibitory receptors (KIR) that, upon engagement, dampen the immune response. CTLA-4 predominantly regulates T-cells at the priming phase of activation by competing with CD28^+^ for binding of B7-1 and B7-2 on DC. CTLA-4 engagement prevents T-cells from achieving full activation. Accordingly, anti-CTLA-4 mAb were shown to activate CD4^+^ and CD8^+^ effector T-cells both directly by removing inhibitory checkpoints and indirectly via the inhibition of regulatory T-cell activity [158]. Eleven EOC patients, previously vaccinated with GM-CSF and irradiated autologous tumor cells, received anti-CTLA-4 ipilimumab (Yervoy^®^, Bristol-Myers-Squibb, BMS). Significant antitumor effects were observed in a minority of these patients and were correlated with increased CD8^+^ T-cells/Treg ratio [159]. In contrast to CTLA-4, PD-1 signaling occurs in the tumor, where PD-L1-expressing tumor cells can signal through PD-1 on TIL to turn-down the antitumor T-cell response. In EOC, the PD-1/PD-L1 pathway seems to be a dominant immunosuppression mechanism [73]. PD-L1 expression in EOC was demonstrated to be an independent unfavorable prognostic factor and to promote peritoneal dissemination [24,160]. Several PD-1/PD-L1-pathway blocking agents were assessed in various cancer types and promising results were recently reported. Nivolumab (BMS-936558, Bristol-Myers-Squibb) was tested in 296 patients most harboring lung cancer, renal cell cancer and melanoma, with clinical benefits apparent in 20 to 25% of the patients [161]. Impressive durable responses were reported. 25/42 patients with PD-L1-positive tumor experienced an objective response while none of the 17/42 PD-L1-negative patients did. However, lack of prognostic association was reported elsewhere and the usefulness of PD-L1 as a biomarker need to be explored in larger prospective studies [162]. In addition, Bramher and colleagues reported that 1/17 EOC patients treated with anti-PD-L1 mAb (BMS-936559) experienced an objective response [163]. New trials enrolling patients with solid tumor of multiple origins are underway and informative data in EOC are expected [164]. Inhibition of the cytotoxic properties of NK-cells through KIR engagement may also contribute to the tumor escape. Some anti-KIR antibodies, such as lilirumab (Bristol-Myers-Squibb), recently entered clinical development phases. First data were obtained in hematological diseases and phase I studies recruiting patients with solid tumors are ongoing [165,166]. As a corollary, agonistic agents of costimulatory molecules such as glucocorticoid-induced TNFR (GITR), OX40, CD137 are candidates to boost the antitumor immune response. A dose-escalation phase I clinical trial (NCT01239134) with agonist anti-GITR mAb (TRX518) was recently initiated.

Third possibility is to repress the activity of enzymes (IDO, ARG-1, iNOS) that were shown to inhibit the immune response. Data from first clinical trials using IDO inhibitors, notably the isomers of 1-methyl-tryptophan (1MT), were disappointing, but these studies may suffer from lack of potent and selective IDO inhibitors. New compounds recently entered clinical trials [167]. A phase II study of IDO inhibitor INCB024360 is currently recruiting patients with biochemical-recurrent-only EOC following complete remission with first-line chemotherapy (clinical trial: NCT01685255). In addition, inhibitors of phosphodiesterase(PDE)-5, e.g. sildenafil, were reported to increase intracellular concentrations of cGPM, resulting in the inhibition of both ARG-1 and iNOS. PDE-5 inhibitors along with nitroaspirin or specific ARG-1/iNOS inhibitors might provide new therapeutic strategy to recover potent antitumor immune response [154].

Lastly, PGE2 was shown to be a crucial immunosuppressive factor in EOC, as it impairs the cytotoxic properties of effector cells such as Vγ9Vδ2T-cells [45] and also induces the differentiation of MDSC from bone marrow stem cells in a mouse model [168]. PGE2 biosynthesis is regulated by the inducible COX-2 enzyme and could be inhibited by the COX-2-specific inhibitor celecoxib (Celebrex^®^, Pfizer). In a mouse model, celecoxib prevented the local and systemic expansion of MDSC, impaired the suppressive function of these cells, and significantly improved vaccine immunotherapy [169]. Thus, celecoxib, currently used in the prevention of colorectal adenomatous polyps [170], could be tested in combination with immunotherapy to reduce the immunosuppression by MDSC in EOC. Another possible strategy to counteract the immunosuppressive influence of PGE2 on Vγ9Vδ2T cells could be to restore the cytotoxic properties of these cells with a zoledronate perfusion [45]. In addition, zoledronate was shown to prevent the immunosuppressive polarization of TAM [171,172] which is a major component of the leukocyte infiltrate in the tumor microenvironment and plays a dominant role in the production of immune suppressive cytokines in EOC [60]. Thus, zoledronate, which is currently used for the management of osteoporosis and bone metastasis, appears to be an attractive molecule to reinforce the immune response. Altogether, these data warrant further exploration of combinatorial therapies with immunotherapy and bisphosphonates.

In conclusion, accumulated evidences support the immunoediting hypothesis and the idea that EOC is immunogenic. Immunotherapeutic protocols aimed at modulating the immune system to strengthen the spontaneous antitumor immune response are under investigation. Targeting the immunosuppressive mechanisms could be the key to fully unleash the potential of immunotherapy. The combination of molecules endowed with immuno-modulatory properties with immunotherapy targeting the tumor cells will hopefully increase the survival of EOC patients. Careful preclinical evaluation will be necessary to screen optimal combinations before clinical trials.

## Competing interests

The authors declare that they have no competing interests.

## Author’ contributions

VL, AT, FF, SH, VJ and FC reviewed the literature; VL, AT and FC wrote the paper; VL, JL, VC, MABR and FC proofread the final copy. All authors read and approved the final manuscript.
